# Cellular Senescence: Many Roads, One Final Destination

**DOI:** 10.1100/tsw.2010.68

**Published:** 2010-04-13

**Authors:** Raya Saab

**Affiliations:** Children's Cancer Center of Lebanon, American University of Beirut, Lebanon

**Keywords:** senescence, tumor suppression, p53, Rb, DNA damage, heterochromatin, oncogene

## Abstract

Cellular senescence is a tumor-suppressor mechanism that has been shown to occur in response to multiple signals, including oncogenic stress, DNA damage, oxidative stress, telomere shortening, and other tumor-promoting insults. Over the past decade, much has been uncovered regarding the phenotype of this tumor-suppressor response and the underlying pathways necessary for its establishment. However, we have also learned that the intricate details of signaling pathways underlying senescence as a tumor-suppressor response are very much context dependent. In addition, cross-talk among pathways, and negative and positive feedback loops, all complicate our understanding of this process. This short review attempts to summarize what is known to date regarding senescence in tumor suppression, both *in vitro* and *in vivo*. Further insights into pathways necessary for senescence will hopefully identify appropriate targets for interventions to not only induce senescence as a treatment of cancerous lesions, but also to maintain this state in premalignant lesions in an effort to prevent progression to cancer.

## WHAT IS SENESCENCE?

The phenomenon of cellular senescence was first described by Hayflick and Moorhead as a cell culture observation, where human diploid fibroblasts irreversibly exit the cell cycle after undergoing serial passage *in vitro*[1]. This “replicative senescence” was found to be primarily related to telomere attrition, and differs from quiescence in that cells exit the cell cycle despite continued mitogenic stimulation and remain metabolically active[2,3]. More recently, it was recognized that cellular senescence also occurs in response to oncogenic stimulation, as well as myriad other stimuli that have the potential of causing cellular transformation. Senescence can be induced by oncogenic signaling, DNA damage, telomere shortening, loss of key tumor suppressors, and oxidative damage[4,5,6,7]. Evidence of senescence was found in premalignant lesions in mouse and in humans, and was not seen in fully transformed counterparts of these lesions (see below)[8]. Because of all the above findings, cellular senescence is now accepted as a tumor-suppressor response that serves to limit expansion of pretumorigenic cells, leading to irreversible cell cycle arrest despite continued mitogenic signaling.

Because features of senescence are seen in premalignant lesions and are lost in malignant tumors, it is thought that senescence is a tumor-suppressor response occurring in cells predisposed to malignancy. This results in cell cycle arrest and the pretumorigenic cells may then remain “dormant” as such. However, since premalignant lesions by definition may progress to cancer, pathways to overcome this tumor-suppressive response must occur. In fact, it is likely that a few cells in a premalignant, largely senescent, lesion either can (1) bypass the senescent state, never undergoing senescence because they have acquired mutations in key genes necessary for the senescence response, or (2) revert from or “escape” the senescent state, possibly due to a low threshold to re-enter the cell cycle upon further genetic insults (see Fig. 1). This latter scenario, although not supported yet *in vivo*, is derived from findings in cell culture models[9]. Our recent work using a mouse model of a Cyclin D1–driven premalignant pineal tumor supports the first scenario. In this model, the Rb pathway is disrupted by transgenic expression of Cyclin D1 in cells of the pineal gland, a neuroendocrine organ in the brain involved in coordinating the circadian rhythm. In these *Irbp-Cyclin D1* mice, pineal cells undergo excessive proliferation that ends in senescence that is both p53 and Rb dependent (Fig. 2A). In the absence of p53, *Irbp-Cyclin D1* cells never undergo senescence and the mice die of an invasive brain tumor, pineoblastoma. When an Rb pathway activator, p18Ink4c, is absent, most of the cells still undergo senescence, but a few cells seem to “bypass” the senescence response that occurs in all other cells and eventually lead to invasive pineoblastoma[10]. Which process occurs in human premalignant lesions that ultimately progress to cancer is yet unclear and may very likely depend on the molecular pathogenic mechanisms leading to tumor progression in each case.

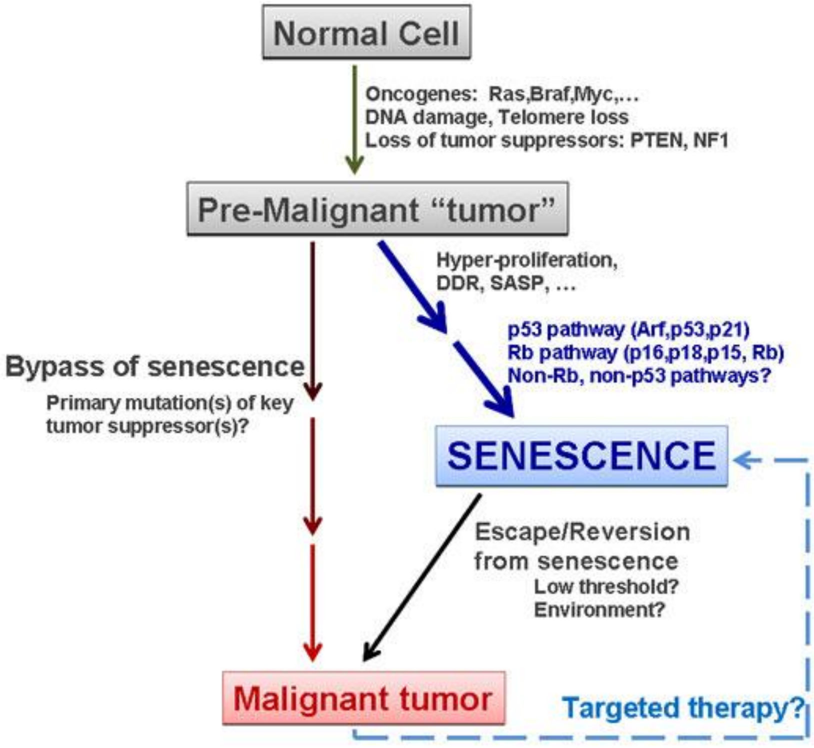

**FIGURE 1.** Premalignant lesions may progress to cancer by either bypass of senescence through disruption of signals responsible for senescence induction or they may escape from an established senescent state by disrupting signals responsible for maintenance of senescence. Identification of key signaling pathways may allow targeted therapy to re-establish senescence in malignant cells.

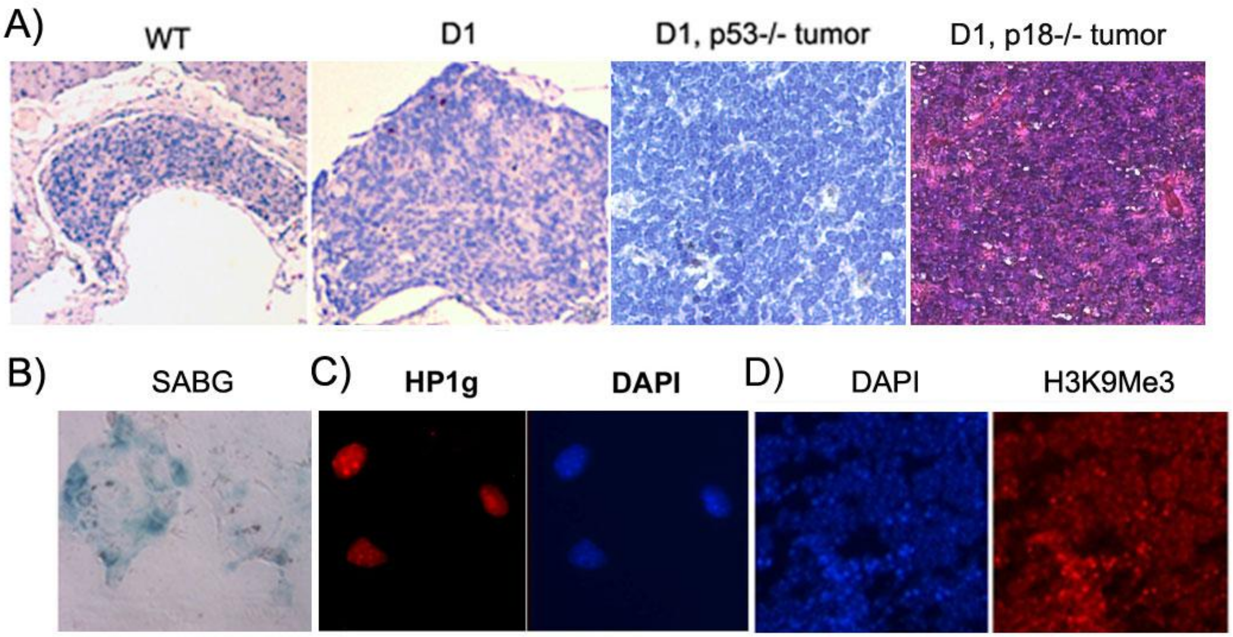

**FIGURE 2.** Features of senescence *in vivo* and *in vitro*. (A) H&E-stained sections of pineal glands from wild-type (WT), transgenic *Irbp-Cyclin D1* (D1) mice expressing Cyclin D1 in the pineal cells, resulting in premalignant "senescent" hyperplastic lesions, and pineal tumor progression in D1 mice in the absence of p53 (D1p53–/–) or p18Ink4c (D1p18–/–). (B) Staining of cultured *Irbp-Cyclin D1* pineal cells with β-galactosidase at acidic pH shows SABG-positive staining consistent with a senescent phenotype. (C) SAHF, shown by staining for HP1γ in *Irbp-Cyclin D1* pineal cells in culture and (D) *in vivo* by staining for H3K9-me3 foci, in *Irbp-Cyclin D1* pineal gland section.

## HOW CAN SENESCENCE BE DETECTED?

One of the barriers to identifying the magnitude and relevance of senescence to human premalignant lesions is that we currently recognize senescent cells based on markers that may not be relevant to all senescent phenotypes. This is because most of the current markers of senescence rely on (1) morphologic/biochemical features of senescent cells in culture and (2) components of signaling pathways involved in the senescence response, which can vary depending on the originating insult and possibly cell type (see below).

### Senescence-Associated β-Galactosidase (SABG) Activity

Senescent cells in culture are known to assume a flattened morphology; however, this is not a marker that can be used *in vivo*. The first marker of senescent cells to gain wide acceptance was the SABG activity[11], where senescent cells, both *in vitro* and *in vivo*, were noted to stain positive for β-galactosidase under acidic conditions (pH 6.0) (Fig. 2B). This positive staining is thought to be due to accumulation of lysosomal β-galactosidase in senescent cells[12,13]. However, this assay is neither sensitive nor specific; it may be positive in nonsenescent cells and sometimes may be negative despite other features of senescence[14]. Thus, although SABG staining can be a reliable marker of senescence, it has to be taken in the right context and in the presence of other conditions consistent with a senescent phenotype, such as cell cycle exit in the presence of mitogenic signaling and other markers of senescence.

## Senescence-Associated Heterochromatin Foci (SAHF)

Senescent cells in culture were noted to contain foci of heterochromatin seen by focal DAPI staining within nuclei[15]. These foci are enriched in repressive marks, such as heterochromatin-associated protein 1-gamma (HP1γ) and histone 3 trimethylated at lysine 9 (H3K9me3)[15]. This phenomenon was first described *in vitro*, and has since been recognized in senescent premalignant cells *in vivo* (Fig. 2C, D). The occurrence of heterochromatin foci in senescent cells likely is responsible for the “irreversible” nature of the senescent state. It is thought to result in suppression of transcription of cell cycle machinery proteins, including the E2Ff family of transcription factors. In RAS-induced senescence, for example, heterochromatin proteins are recruited by the retinoblastoma (RB) protein to E2F-responsive promoters to form SAHF[15]. We also found that by using gene expression analysis, SAHF-containing pineal cells from 2-month-old *Irbp-Cyclin D1* mice differ from nonsenescent (*Irbp-Cyclin D1, p53–/–*) pineal cells primarily in expression of cell cycle–associated genes (unpublished data). Although this could be a secondary phenomenon due to cell cycle progression in *Irbp-Cyclin D1, p53–/–* cells, it may well be the result of direct suppression of cell cycle genes by heterochromatin foci in senescent cells. Because SAHF represent an end mechanism through which the senescence-associated transcriptional repression is thought to be maintained, it has become a rather reliable marker of the phenotype of senescent cells, both *in vitro* and *in vivo*[16].

## Cell Cycle Inhibitors

Expression of certain cell cycle inhibitors, such as p16INK4a, p15INK4b, p14ARF (p19Arf in the mouse), p21Cip1, and others, has been correlated with senescence. However, as the pathways to the senescent end point do seem to differ (discussed below), these proteins remain markers of a particular pathway and may not be positive in all senescent phenotypes. In fact, the expression of these proteins likely depends on the cell type, initiating insult, and possibly the cellular context and microenvironment in which the lesion develops[9,17,18]. These “signaling pathway components” are therefore helpful in identifying senescent lesions where they are involved in the signaling cascade, but their absence does not preclude senescence occurring via an alternate signaling pathway. In addition, their presence cannot be used to identify senescence except in the appropriate context because it is known that these proteins can be overexpressed when mutated or ineffective. For example, in tumor cells where downstream effector proteins are mutated, upstream regulators are frequently overexpressed, but ineffective[19]. Thus, to be reliable senescence markers, they need to be combined with other features of senescence, such as absence of proliferation despite oncogenic/mitogenic signaling.

## Gene Expression Patterns

Senescent cells differ in their gene expression pattern compared to reversibly arrested, quiescent cells[20,21,22]. Gene expression profiling was useful, in fact, in identifying more markers of senescence, which now include proteins like Dec1, Mcl1, TRAIL, and DcR2, both in cultured fibroblasts and in lesions from chemically induced skin cancer and *Ras*-induced lung adenomas in mice[8]. Again, because the role of these proteins in the senescent response is still unclear, their expression needs to be interpreted in a context-specific manner.

## Senescence-Associated DNA Damage Foci (SDF)

Focal staining for proteins involved in the DNA damage response (DDR) has recently been recognized as another feature of senescent cells. DDR signaling, whether due to primary DNA damage or due to replicative stress secondary to oncogenic signaling, results in p53 pathway activation, contributing to establishing the cell cycle arrest and the senescent phenotype[23,24,25]. Components of the SDF implicated in signaling upstream in the senescence response have included γH2AX, 53BP1, pATM, pChk1, and pChk2[25,26,27]. In some tissues, such as colon and bladder, DDR foci are seen in premalignant lesions to a higher extent than in their malignant counterparts. In such cases, DDR pathway activation has been presumed to possibly act as a continuous signal to maintain the senescent state[24,26,28]. However, in other types of precancerous lesions, such as *K-RasV12*–driven lung adenomas, no DDR foci are detectable in senescent lesions[29]. In Cyclin D1–expressing pineal cells in the *Irbp-Cyclin D1* mouse, we observe SDFs early on during Cyclin D1–induced proliferation, but they resolve after senescence has set in (unpublished data), suggesting that although the DDR pathway may be involved in initiating the senescent response, it may not be necessary for maintaining it in this setting. Thus, SDFs are useful markers of senescence when they are combined with lack of proliferation in a premalignant lesion, but their absence again does not preclude a senescent phenotype.

### Senescence-Associated Secretory Phenotype (SASP)

Senescent cells were also found to up-regulate transcription of multiple secreted factors, including extracellular proteases, matrix metalloproteinases, growth factors, proinflammatory cytokines, and chemokines. This cocktail has been referred to as the senescent-associated secretory phenotype (SASP)[30,31,32]. Some of these factors have been shown to promote tumorigenesis in surrounding nonsenescent cells[33], while others have been shown to have tumor-suppressor effects, such as IGFBP7[34]. Others, such as IL6 and IL8, can have either effect depending on context[31,35,36,37]. In addition, many of these secreted proinflammatory cytokines and chemokines are thought to possibly promote immune-mediated clearance of senescent cells as a tumor-suppressive mechanism[38]. Importantly, some of these cytokines are expressed in premalignant human lesions, suggesting relevance to maintaining senescence and tumor suppression[30]. The use of these factors as markers of senescence again should be combined with other features of senescence, due to their having protumorigenic and antitumorigenic effects based on context.

## WHAT IS KNOWN ABOUT SIGNALING PATHWAYS LEADING TO SENESCENCE?

Many of the above markers used to identify senescence are in fact directly involved in signaling leading to senescence. Two major tumor-suppressor proteins, RB1 and p53 (TP53), have been found to be involved, either alone or in combination, in most instances of senescence. Signaling pathways upstream and downstream of these two proteins have been identified as important for senescence in specific settings, as detailed below.

### Ras Pathway

Oncogene-induced senescence *in vitro* was first described in the context of oncogenic stimulation by the activated form of Ras, *HRAS-G12V*[4]. The mammalian *RAS* family consists of three highly conserved genes: *H-RAS*, *K-RAS*, and *N-RAS*[39]. Ras activation triggers a number of downstream signaling pathways, including the Raf/MEK/MAPK pathway[40], the phosphoinositol 3-kinase (PI3K) pathway, and an increase in formation of reactive oxygen species (ROS)[41,42,43,44]. *RAS* mutations have been found in close to 30% of human cancers[45], with common activating Ras mutations, including single amino acid substitutions at residues 12, 13, 59, or 61, leading to oncogenic properties and transformation[46,47].

Serrano et al. first showed that, despite an initial hyperproliferative response, Ras-induced proliferation in normal cells leads to cell cycle arrest and senescence[4]. In addition, the Raf-Mek pathway downstream of Ras was shown to be responsible for the induction of senescence[40]. Since then, several cell culture and mouse models have shown Ras to be a strong inducer of senescence. *In vivo*, endogenous oncogenic *Kras* (*Kras-G12V*) triggers senescence during the early stages of *Ras*-induced lung and pancreatic tumors[8]. Expression of *Kras-G12V* in the mammary gland also causes senescence[48], and *Braf-V600E* induces senescence in lung tumors and melanocytic nevi *in vivo* in mice[49]. In chemically induced skin papillomas, which are mediated by oncogenic activation of HRAS, senescence also occurs, mediated by activation of p38 MAP kinase[50]. In addition to the Ras oncogenes and their proximal downstream kinases, distal effectors of the Ras pathway, such as the E2F family of transcription factors, can also induce senescence[51].

Ras-induced senescence is accompanied by overexpression of both p16INK4a and p19ARF, and the consequent activation of RB and p53[4,18,40,52]. Conversely, in the absence of p16Ink4a, p19Arf, or p53, Ras can induce cellular transformation directly in mouse embryonic fibroblasts (MEFs)[4,53], thus underscoring the importance of these tumor suppressors in the Ras-induced senescence response. Ras pathway activation can lead to senescence via downstream effectors that include the Raf-Mek or p38MAPK pathways, ROS[44,54], DDR[25], and the p53 and Rb pathways, discussed further below (see Fig. 3). Importantly, the extent of activation of the Ras pathway seems to be important for triggering senescence, as low levels of RAS expression do not activate this response as efficiently as do high levels[48].

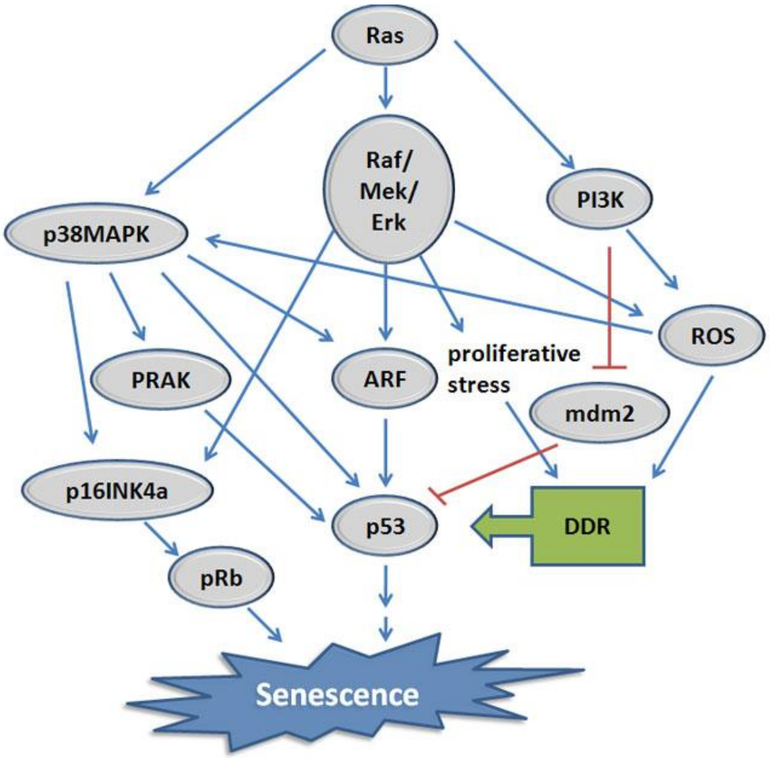

**FIGURE 3.** Schematic diagram of Ras pathway activation leading to senescence. Ras can activate the Raf/Mek/Erk pathway, PI3K pathway, and p38MAPK, leading to downstream signaling, ultimately resulting in p53 and/or Rb activation that mediates the senescent response.

## Cyclins, Cyclin-Dependent Kinases, and Components of the Cell Cycle Machinery

Regulators of the cell cycle, such as Cyclins and Cyclin-dependent kinases (Cdks), are often downstream of oncogenic signaling, and may themselves drive the cell cycle in tumorigenic or pretumorigenic lesions by undergoing activating mutations or gene amplifications[55]. Thus these proteins, when overexpressed or constitutively active, essentially act as oncogenes. In fact, similar to oncogenes like Ras, several of these cell cycle proteins have been shown to induce senescence after a hyperproliferative response. Bartkova et al. first showed that Cyclin E expression leads to senescence in cultured human fibroblasts; they also showed that cdc6 has the same effect[24]. Both seemed to act via inducing a DDR due to hyper-replication and replicative stress. Our studies showed that Cyclin D1 expression in photoreceptor progenitor cells in the neuroendocrine pineal gland results in hyperproliferation limited by both p53- and Rb-dependent senescence[10]. Loss of either p18Ink4c (an activator of the Rb pathway by Cdk4 inhibition), or p53, caused progression to invasive pineoblastoma.

A recent study showed that Cdk2 acts to suppress Myc-induced senescence[56]. Myc-induced senescence occurred in *Cdk2*$^{-/-}$ cells, but not in *Cdk2*$^{+/+}$ cells, and was due to activation of the p53 and Rb pathways, with induction of p21CIP1 and p16INK4a. Loss of Cdk2 promoted Myc-induced senescence in pancreatic β-cells and splenic B-cells *in vivo*, and delayed lymphoma onset in the latter. In addition, pharmacological inhibition of Cdk2 induced Myc-dependent senescence in various cell types, including a *p53*-null human cancer cell line. In the *Irbp-Cyclin D1* mouse, we have observed marked down-regulation of Cdk2 upon cell cycle exit in Cyclin D1–driven senescence of pinealocytes (unpublished data). These findings, in addition to the elegant studies by Campaner et al. above, suggest that Cdk2 suppression may in fact act downstream of p53 in the pathway to senescence, leading to activation of the Rb pathway and possibly linking the Rb and p53 pathways in the senescence response. This, however, is speculative at this point and needs to be further investigated.

## Loss of Tumor Suppressors

Several instances of senescence precipitated by loss of tumor suppressors, rather than oncogenic activation, have been described. This probably occurs due to an imbalance in signaling favoring oncogenic stimulation in some cases. For example, deletion of the tumor-suppressor *NF1* causes senescence by activation of the endogenous Ras-Erk-Akt pathway, followed by a feedback mechanism leading to down-regulation of this pathway[57]. Another example of senescence triggered by loss of a tumor suppressor occurs in mice with conditional deletion of the Von Hippel–Lindau (*Vhl*) gene in kidney cells[58]. Thus, it is likely that oncogenic signaling, whether by direct oncogene activation or indirectly through abolishment of tumor-suppressor feedback responses, commonly underlies these lesions in inducing a senescent response. However, this is not always true: Inactivation of PTEN, a phosphatase that opposes PI3K/Akt activity, in mouse prostate leads to prostate intraepithelial neoplasia (PIN) with features of cellular senescence[6]. Similarly, targeted expression of Akt1 in the prostate leads to the formation of PIN lesions with cellular senescence. However, in PTEN-deficient cells, senescence seems to be different than that induced by oncogenes. Specifically, senescence in this setting is not dependent on a hyperproliferative phase, p19Arf, or a DDR[59].

## DDR Pathway

As introduced above, the DDR machinery is now recognized to be a mediator of senescence due to many insults that result in DNA damage signaling, including telomere attrition, oncogenic signaling leading to hyper-replication, ROS damage, and others[24,25,60,61,62]. In human cell culture models of activated Ras, Mos, cdc6, Cyclin E, stat 5, and in *Ras*-driven mouse tumors, a DDR is triggered and is necessary for signaling to p53-dependent senescence[24,25]. This activation of the DDR by oncogenes is thought to be the direct result of replication stress due to excessive proliferative signals[25], and abrogation of components of the DDR pathway abolishes p53-dependent senescence in, for example, Ras-induced tumors[24,25]. In addition, several human premalignant lesions, including melanocytic nevi, dysplastic and adenomatous lesions in the breast, colon, and bladder, all show evidence of DDR pathway activation[26]. It is therefore likely that during tumor progression, cells with an abrogated DDR pathway are selected for, leading to continued proliferation and escape from senescence. In such a scenario, targeting these deregulated DDR pathways may then offer new therapeutic approaches in the treatment of such tumors, via senescence induction.

## Reactive Oxygen Species (ROS)

Previous studies have shown that mitogenic signaling can result in increased levels of intracellular ROS[63]. In cell culture models of oncogene-induced senescence, an increase in the levels of ROS was found to be associated with senescence induction and maintenance, and treatment with oxygen radical scavengers abolished the senescent response[54,64,65,66]. Interestingly, ROS production was found to be augmented by a positive feedback loop involving the p16Ink4a-Rb pathway, as well as the activation of protein kinase Cδ (PKCδ)[67]. Although ROS have been shown to cause DNA damage and DDR activation[68], whether ROS signals via the DDR pathway in senescence induction has not yet been adequately investigated. In fact, the relative contribution of ROS and DDR to senescence induction and maintenance, and whether they act in series or in parallel in different contexts, is currently still unclear.

The role of hypoxia in senescence is less clear. Standard cell culture conditions expose cells to relatively hyperoxic conditions, resulting in ROS production, which in turn is thought to contribute to senescence induced by serial culture *in vitro*[69,70]. Tissues *in vivo* are exposed to physiological oxygen tensions, ranging from ~2 to 14% depending on the tissue; this is commonly termed “physiologic hypoxia” because it is lower than the atmospheric oxygen that cultured cells are exposed to in standard conditions[71]. Studies have shown that a decrease in oxygen tension to physiologic levels can in fact delay or abrogate cell culture–induced senescence[54,70]. Recently, a role has been uncovered for the hypoxia-inducible factor 1α (Hif1α) in the senescence response. Hif1α is a transcriptional regulator that is activated in response to hypoxia and controls hypoxic cellular responses, such as glycolysis, erythropoeisis, and angiogenesis[72,73]. Although Hif proteins are expressed and active at physiologic oxygen conditions, levels are increased by both protein stabilization and increased transcription when oxygen levels are low[73,74,75].

Loss of Hif1α sensitizes cultured MEFs to replicative senescence, and promotes proliferation at “physiologically hypoxic” oxygen levels[76]. However, whether and how Hif1α contributes to the senescence response *in vivo*, in response to the much smaller fluctuations in oxygen levels between normal and tumor tissue, is still to be determined. For example, there is evidence that ROS produced by mitochondria in response to hypoxia are in fact necessary and sufficient for stabilization of Hif[77,78,79,80]. Reconciliation of these findings will require further understanding of the interplay of these pathways under physiologic conditions *in vivo*.

The tumor-suppressor protein Von-Hippel Lindau (pVHL) is involved in the response to hypoxia, primarily directing the polyubiquitylation and proteosomal degradation of HIF1α under well-oxygenated conditions[81,82]. pVHL also has HIFα-independent functions[83]. Recently, VHL was also found to play a role in the senescence tumor-suppressor response. Acute inactivation of VHL causes a senescent-like phenotype *in vitro* and *in vivo*, independently of Hif[58]. Senescence in this setting was independent of p53, but depended on Rb activation via up-regulation of the Cdk-inhibitor p27. In addition, down-regulation of the SWI2/SNF2 chromatin remodeler p400 was essential for senescence induced by VHL loss, thus linking chromatin remodeling to VHL-related senescence. Although the senescence response to VHL loss is independent of Hif, whether it is related to hypoxia-related responses regulated by VHL is unclear.

## Inflammatory Cytokines and Components of the SASP

Components of the SASP play a role in both induction and maintenance of senescence in an autocrine manner. It was shown that knockdown of CXCR2 (which is a receptor for IL8 and CXCL) leads to bypass of senescence in human diploid fibroblasts, and overexpression of CXCR2 causes p53-dependent senescence[30]. It was also found that IL-6 and IL-8, and their receptors, play a causative and necessary role in the establishment and maintenance of senescence[30,31]. However, as mentioned above, despite the autocrine tumor-suppressive effect of these factors, paracrine effects promoting tumorigenic properties have been described[36,37].

## RB and p53 Tumor-Suppressor Pathways

Most studies to date have shown senescence to be dependent on either the RB or p53 pathway, or both. In MEFs, loss of p53 or inactivation of the Rb family of proteins was sufficient to bypass cellular senescence[84,85]. RAF-induced senescence in human fibroblasts occurs independently of p53, but via p16INK4A and pRB[18]. In contrast, in mammary epithelial cells, RAF induces senescence independently of both p53 and p16INK4a[17].

These observations suggest that p53 and RB may form a single linear pathway in some contexts, while inactivation of both is needed for bypass of senescence in others. In Cyclin D1–driven senescence in the pineal gland, we found that the Rb and p53 pathways were activated at different times during the senescence process; interestingly, p53 activation was transient while RB activation was delayed, but stable. Inactivation of either pathway, however, was sufficient for senescence bypass and tumor progression[10]. It therefore seems that the relative contributions of p53 and RB to cellular senescence vary with the initiating stress/tumorigenic events, cell type, and cellular context.

In addition, although initial studies implicated either ARF or p21CIP1 in p53-dependent senescence, and p16INK4a in RB-dependent senescence, in other settings these mediators were dispensable, suggesting other effectors of senescence[9,17,18,86]. In fact, it is likely that both cell type– and insult-specific differences dictate the signaling through which senescence is induced. Other Cdk inhibitors may be involved in different settings; for example, p15INK4b, rather than p16INK4a, is involved in BRAF-V600E–driven senescence[31], and p27 is essential for senescence induced by VHL loss[58]. Our work shows that neither Arf nor p16Ink4a is involved in Cyclin D1–driven senescence in the mouse pineal gland; in contrast, p18Ink4c and p27Cip1 are up-regulated (activators of the Rb pathway), and the DDR is activated and therefore may be involved upstream of the p53 pathway([10] and unpublished data).

Thus, the roles of the RB and p53 pathways, as well as the relative contributions of their specific upstream and downstream effectors, to the senescence response seems to be subject to cell type–specific and context-dependent differences. Further studies are needed to delineate whether compensatory and redundant mechanisms exist among these multiple paths to senescence, for use in devising therapies for tumor prevention and control.

## Mediators of Heterochromatin Condensation

As mentioned above, SAHF have been observed as a feature of senescent cells and are thought to be responsible for the irreversible arrest of these cells due to transcriptional repression of cell cycle genes. Formation of these SAHF has now been shown to depend on an active RB pathway[15,87]. In fact, pRB was shown to colocalize directly with SAHF[15] and inactivation of the p16INK4a/RB pathway impairs formation of *RasG12V*-induced SAHF in IMR-90 human fibroblasts[15]. On the other hand, the role of p53 in formation of SAHF is still controversial. Early studies showed that a dominant-negative p53 that abolishes p21 activation does not affect *RasG12V*-induced SAHF formation[15], but other studies since then showed that the p53 pathway is necessary for formation of SAHF in other contexts[87,88]. In fact, it is likely that RB and p53 form distinct pathways leading to SAHF formation, and that their relative importance differs with the cellular context. As discussed above, whether RB and p53 act in parallel or intersecting processes is still unclear and may be context dependent.

Over the past few years, there have been significant insights into the mechanisms of formation of SAHF. SAHF contain several common markers of heterochromatin, including H3K9Me3 and bound HP1 protein. SAHF are also characterized by their depletion of linker histone H1 and enrichment in macroH2A and HMGA proteins[89,90,91]. In fact, while HP1 may be dispensable, HMGA1 is required for formation of SAHF[90]. Other proteins are involved in forming SAHF, including PML, HIRA, and ASF1a[90]. The specifics of the interplay between these proteins and the activated RB and p53 pathways in executing the senescence response are still to be determined.

Downstream effectors contributing to SAHF formation include SUV39H1, a histone methyltransferase that is responsible for H3K9 methylation[92]. This enzyme has been shown to bind to RB, further implicating the RB pathway in direct execution of SAHF[93,94]. Other proteins involved in chromatin remodeling, such as p400 and BS69, have also been implicated in p53-dependent senescence[88,95]. Several methyltransferases have recently been found to contribute directly to gene silencing related to senescence. The H3K27-specific methyltransferase EZH2 is lost from the Ink4a/Arf locus as cells undergo senescence[96], concomitant with increased transcription of these genes. Another DNA methyltransferase, JMJD2C, has been found to be associated with heterochromatin foci containing H3K9Me3. Expression of this demethylase leads to a decrease in heterochromatin foci, and inhibition leads to increased proliferation *in vitro*[97]. Interestingly, a recent study using high-resolution SNP genotyping of pediatric medulloblastoma samples identified mutually exclusive mutations targeting genes involved in histone methylation in a total of 19% of cases[98]. Whether such mutations in genes involved in epigenetic modifications are common in other types of cancer is still to be determined.

Of note, data are accumulating showing interplay among the different signals contributing to senescence, and suggesting the presence of multiple feedforward and feedback loops among them. For example, an activated DDR pathway seems to trigger secretion of SASP factors[27], accumulated ROS induces the DDR pathway[68], and activation of the p16Ink4a/RB pathway results in ROS induction via a feedback loop that enhances the senescent phenotype[67]. Future studies will hopefully shed more light on the inter-relationships and interplay of these senescence-associated signaling networks; it would not be surprising if the final product of a senescent phenotype depends on a variety of such cross-talks that differ depending on cell-, microenvironment-, and insult-specific contexts.

## HOW CAN SENESCENCE BE USEFUL IN CLINICAL ONCOLOGY?

As discussed above, evidence of senescence has been observed in various human premalignant lesions, including melanoma, colon and lung adenomas, PIN, and others. The fact that a subset of these lesions eventually progresses to malignant invasive tumors suggests that, in some lesions, premalignant cells either bypass or escape the senescent response, leading to tumor progression (Fig. 1).

Understanding the mechanisms underlying this progression will be important for novel clinical interventions towards cancer prevention and therapy. For example, critical mediators that would need to be inactivated for evasion or escape from senescence may serve as markers to identify lesions at high risk of malignant progression. This would be useful for clinical decisions regarding management of such lesions. Gauthier and colleagues recently showed that for premalignant breast lesions (ductal carcinoma *in situ*, DCIS), markers that suggest a senescent phenotype (high p16Ink4a and low Ki67 expression) may identify women at a lower risk for tumor progression[99]. Such stratification may allow pursuit of less aggressive therapeutic options for patients with low-risk lesions, or alternatively more aggressive interventions for those with high-risk premalignant lesions.

Targeting the pathways involved in senescence may also prove useful as a preventive or therapeutic approach to either maintain or re-establish senescence in clinical premalignant and malignant lesions, respectively. For example, inducible antagonization of Tbx2, which acts to suppress senescence by down-regulating ARF and p21WAF1, was shown to restore senescence in melanoma cells in culture[100,101]. More recently, Alimonti and colleagues[59] showed that cellular senescence in *Pten*$^{+/-}$ MEFs is potentiated by pharmacologic inhibition of the remaining PTEN protein using a novel inhibitor. They also showed that pharmacologic inhibition of PTEN can lead to senescence and inhibit tumorigenesis of human prostate cancer xenografts, using cell lines that express low levels of PTEN and have intact p53. Because senescence in *Pten*$^{+/-}$ cells is p53 dependent, Alimonti and colleagues also demonstrated that therapies directed at p53 stabilization can be effective. Their work shows that Nutlin, through stabilization of p53, dramatically enhances the senescence response of *Pten*$^{-/-}$ MEFs *in vitro*, and enhances senescence and decreases prostate tumor size in *Pten*$^{-/-}$ mice *in vivo*. As mentioned above, the investigators also showed that senescence in *Pten*$^{-/-}$ cells differs from that induced by oncogenes, in that it is not dependent on a preceding hyperproliferative phase, p19Arf, or a DDR[59]. This is important because it underscores the fact that translational approaches to senescence induction and maintenance will need to take into account the particular pathways underlying this tumor-suppressor response in different tissues and contexts. As we learn more about the key signaling effectors involved in specific premalignant lesions, the hope is that appropriate targets will be identified for preservation and/or reinduction of senescence in the various human tumors.

## CONCLUDING REMARKS

Despite the many insights into tumor-suppressor pathways underlying senescence over the past decade, there is still much that needs to be clarified. For example, we still are not certain whether the signals that initiate senescence and those that maintain senescence are separate or whether they overlap in human premalignant lesions. We have just started to appreciate the influence of the microenvironment on the senescence response; the fact that oncogenic signaling results in proliferation and formation of premalignant hyperplastic lesions that are only later followed by senescence suggests the presence of other, noncell-autonomous factors that might help initiate the cell cycle arrest. As mentioned above, it is still unclear whether the progression of premalignant lesions to invasive cancer occurs by primary bypass of senescence in a few cells, or whether cells that have senesced can “escape” this tumor suppressor response due to cellular or microenvironment changes. Finally, recent data[102] suggest that senescent cells can influence their microenvironment to promote malignant transformation of neighboring normal cells, necessitating a very careful approach to the use of senescence-inducing therapies in prevention and therapy of human cancers, as the safety of such therapies will need to be established.
